# Toward actionable biomarkers in psychiatry: a collaborative roadmap for precision clinical trials. An ACNP position paper

**DOI:** 10.1038/s44277-026-00069-w

**Published:** 2026-09-18

**Authors:** Sahib S. Khalsa, Deanna Barch, Linda S. Brady, Teresa Buracchio, William A. Carlezon, Christopher Chatham, Wayne C. Drevets, C. Neill Epperson, Amit Etkin, Bernard Fischer, Danielle L. Graham, Steven C. Hoffmann, Bashkim Kadriu, Martien J. H. Kas, Hartmuth Kolb, Marion Leboyer, Sarah H. Lisanby, Lauren Liss, Husseini K. Manji, Valentina Mantua, Hugh M. Marston, William Potter, Frank Yocca, Kerry J. Ressler

**Affiliations:** 1 Department of Psychiatry and Biobehavioral Sciences, Semel Institute for Neuroscience and Human Behavior, David Geffen School of Medicine, University of California, Los Angeles, CA USA; 2 Department of Psychological and Brain Sciences, Washington University in St. Louis, St. Louis, MO USA; 3 Department of Psychiatry, Washington University School of Medicine, St. Louis, MO USA; 4 National Institute of Mental Health, Bethesda, MD USA; 5 Office of Neuroscience, Office of New Drugs, Center for Drug Evaluation and Research, United States. Food and Drug Administration, Silver Spring, MD USA; 6 McLean Hospital, Mass General Brigham Department of Psychiatry, Harvard Medical School, Cambridge, MA USA; 7 Shionogi, Inc, Florham Park, NJ USA; 8 Johnson & Johnson, San Diego, CA USA; 9 Department of Psychiatry, University of Colorado School of Medicine, Anschutz Medical Campus, Aurora, CO USA; 10 Alto Neuroscience, Inc., Mountain View, CA USA; 11 Stanford Department of Psychiatry and Behavioral Sciences, Stanford University, Stanford, CA USA; 12 Department of Biomarkers and Systems Biology, Biogen, Cambridge, MA USA; 13 Foundation for the National Institutes of Health, North Bethesda, MD USA; 14 Bristol Myers Squibb, Lawrence, NJ USA; 15 Groningen Institute for Evolutionary Life Sciences, University of Groningen, Groningen, the Netherlands; 16 Fondation FondaMental, Créteil, France; 17 Translational Neuropsychiatry Laboratory, Inserm U955, Paris-Est Créteil University, Créteil, France; 18 Department of Psychiatry, Mondor University Hospital, AP-HP, Créteil, France; 19 Arizona State University John Shufeldt School of Medicine and Medical Engineering, Phoenix, AZ USA; 20 Neuroscience and Mental Health Discovery Research, Boehringer Ingelheim, Biberach, Germany; 21 Oxford University, Oxford, United Kingdom; 22 Yale University, New Haven, CT USA; 23 UK Government Mental Health Mission, London, United Kingdom; 24 Independent Expert, Philadelphia, PA USA; 25 BioXcel Therapeutics Inc, New Haven, CT USA

**Keywords:** Emotion, Biomarkers

## Abstract

Psychiatric drug development remains constrained by diagnostic heterogeneity, insufficient and inconsistent drug response, high placebo response rates, and the absence of validated biomarkers that can reliably guide patient stratification, trial design, and regulatory decision-making. Despite substantial advances in neuroscience, few biomarkers in psychiatry have progressed beyond exploratory use, limiting the translation of mechanistic insights into effective therapeutics. The process of implementing biomarkers in psychiatry requires new levels of organization and coordination across many stakeholders, each with their own priorities. To date, however, no comprehensive roadmap exists to align scientific, regulatory, clinical, and commercial stakeholders around the development and implementation of predictive biomarkers in psychiatry. This position paper, developed by the American College of Neuropsychopharmacology (ACNP) Precompetitive Stakeholder Task Force and informed by work from the European College of Neuropsychopharmacology (ECNP) on the Precision Psychiatry Roadmap, brings together key stakeholders to begin the process of engaging in coordinated efforts. By integrating scientific, regulatory, and commercial perspectives, we clarify biomarker definitions and contexts of use, review current FDA regulatory pathways and gaps, and synthesize lessons from real-world examples. We highlight emerging biomarker modalities including fluid-based, digital, electrophysiological, neuroimaging, and multimodal approaches, and propose strategies to de-risk their adoption through standardized platforms, early regulatory engagement, and scalable trial designs. Emphasis is placed on precompetitive collaboration, large-scale data harmonization, and stakeholder alignment to accelerate reproducibility, regulatory interpretability, clinical utility, and improved use of experimental systems. This roadmap identifies actionable strategies to advance biomarker-enabled precision psychiatry and nurture cooperation, which will accelerate therapeutic innovation and improve clinical outcomes.

## Lay Summary

Psychiatric drug development remains constrained by diagnostic heterogeneity, insufficient and inconsistent drug response, high placebo response rates, and the absence of validated biomarkers that can reliably guide patient stratification, trial design, and regulatory decision-making. Despite substantial advances in neuroscience, few biomarkers in psychiatry have progressed beyond exploratory use, limiting the translation of mechanistic insights into effective therapeutics. The process of implementing biomarkers in psychiatry requires new levels of organization and coordination across many stakeholders, each with their own priorities. This roadmap identifies actionable strategies to advance biomarker-enabled precision psychiatry and nurture cooperation, which will accelerate therapeutic innovation and improve clinical outcomes.

## Introduction

### Background and motivation

Psychiatric drug development continues to face major challenges, including diagnostic heterogeneity, high placebo response rates, and a lack of clearly defined mechanistic targets. Unlike in other therapeutic areas of medicine, clinical trials in psychiatry still predominantly enroll patients based on symptom checklists rather than pathophysiological criteria. For example, psychiatric symptoms such as anhedonia, cognitive dysfunction, and psychomotor slowing are observed across multiple psychiatric disorders and also manifest in neurodegenerative or systemic diseases, such as Parkinson’s disease, stroke, and lupus. This cross-diagnostic overlap complicates the identification of specific clinical populations and impedes the development of targeted treatments [1, 2].

While there have been numerous attempts over the decades to identify biomarkers that could stratify patients based on likely response to specific pharmacologic mechanisms—or even predict placebo responsiveness—these efforts have yielded few clinically actionable tools [3–5]. Commonly investigated biomarkers such as cortisol, C-reactive protein (CRP), and mGluR (metabotropic glutamate receptor) genetic variants showed promise in biomarker discovery studies, but evidence was not replicated in larger interventional trials (Table 1). Others, such as composite panels combining electroencephalogram (EEG), digital, and blood-based measures, remain hypothetical. Large-scale genetic efforts, such as the Psychiatric Genomic Consortium, have had numerous successes in identifying common variants associated with psychiatric disease; however, the identified variants to date do not carry strong predictive value or penetrance for the majority of patients. Note that there have been a limited number of highly penetrant variants with high positive predictive value in some classical neurodevelopmental disorders, as well as more recent data identifying potentially causal rare variants [6], but these genetic markers are only relevant for a very small percentage of the population suffering from psychiatric illness. While combined polygenic risk scores (PRS) may promise future utility in smaller sample sizes or even at the individual level, they currently provide only modest prediction of risk, and they are only recently figuring prominently in clinical trials. Compounding these difficulties is a perceived lack of regulatory clarity and high perceived business risk associated with investing in psychiatric conditions even without the burden of validating novel biomarkers, further stalling translation.

**Table 1:** conceptual framework for biomarker maturity and strategic use in Central Nervous System (CNS) drug development.

Category	Example	Insight
Success	Biomarkers that confirm the presence of amyloid plaques (i.e., PET, CSF, or blood) for patient selection in Alzheimer’s disease [107, 108] (e.g., lecanemab) [7]; Tau PET for enrichment in donanemab [109] Plasma neurofilament light chain (NfL) as a marker of disease progression and surrogate endpoint of clinical benefit in a clinical trial of *superoxide dismutase 1* (SOD1) in ALS [110]	Demonstrates how imaging and fluid biomarkers can be used to confirm target pathology or enrich for treatment responsive subgroups when clearly linked to mechanism and supported by outcome data Highlights that reductions in NfL protein, a marker of neurodegeneration and axonal damage, can be used to infer treatment effect. Also highlights that NfL can be used as a prognostic biomarker of ALS disease progression and survival
Challenging / Hypothetical	C-reactive protein (CRP) [111, 112]; cortisol in major depressive disorder (MDD) [113]; mGluR variants [114] Multimodal MDD stratification panel (digital phenotyping + EEG + blood biomarkers) [115]	Promising biological signals in biomarker discovery studies, but inconsistent replication in interventional trials Conceptually attractive approaches to address heterogeneity, but requires standardization, replication, and regulatory alignment
Strategic Use	EEG predictors of treatment and/or placebo response [116–122]	Predictive potential shown in several studies, yet underutilized in trial design despite potential to improve signal detection

Table 1 presents a conceptual framework illustrating different stages of biomarker maturity and strategic application. Examples are intended to be illustrative and do not imply formal regulatory qualification unless explicitly stated.

In contrast, the fields of Alzheimer’s disease (AD), Parkinson’s disease (PD), and amyotrophic lateral sclerosis (ALS) may offer compelling examples of how large-scale, unbiased, longitudinal studies, coupled with biospecimen repositories and collaborative infrastructures, have catalyzed the emergence of useful stratification frameworks, validated biomarkers, and novel hypotheses for both clinical and preclinical research. Building on the success of Alzheimer’s disease biomarkers and ongoing drug development in other neurological conditions (PD, ALS, Duchenne muscular dystrophy), regulatory pathways have become increasingly clear and actionable in the neurodegeneration space. For example, the use of amyloid pathology as a patient inclusion biomarker has significantly transformed the landscape of clinical trials in AD. This innovation was followed by technological advances and in vitro diagnostic (IVD) approvals, which enabled the use of fluid-based tests—analyzing cerebrospinal fluid (CSF) or blood that demonstrated high concordance with amyloid positron emission tomography (PET)—to identify patients with amyloid pathology. These advances ensure that only patients with the pathology under investigation are enrolled in trials, thereby enhancing the precision and reliability of study outcomes. Notably, the FDA labels for lecanemab (Leqembi®) and donanemab (Kisunla®) instruct clinicians to confirm amyloid beta pathology prior to initiating treatment. The Kisunla® label describes tau PET-defined subgroups in the clinical trial evidence base, although tau PET is not listed as a required patient-selection criterion in the label (see FDA label documents, 2023 [7]).

These precedents suggest that, even in clinically heterogeneous populations, large, collaborative biomarker programs—when designed with appropriate standardization, data sharing, and analytic rigor—can produce reproducible insights and testable mechanistic hypotheses (e.g., Table 2). Similar strategies are needed in psychiatry to catalyze a precision medicine approach. Although psychiatric disorders currently lack a well-defined common neuropathology, technological advances in brain imaging, wearable biosensors, transcriptomics, proteomics, and computational modeling provide the necessary tools to construct a translational biomarker toolbox. Such a toolbox would enable researchers to (a) identify human analogs of brain-based functional changes observed in preclinical models, and (b) define more homogeneous biotypes across psychiatric symptoms and diagnoses [1, 2]. Ultimately, this could pave the way for the identification of diagnostic and predictive biomarkers and the definition of novel biology-based populations amenable for targeted treatments.

**Table 2:** Examples of pre-competitive consortia focused on biomarker discovery in CNS drug development.

Program	Area of Research	Disease / Indication
FNIH Biomarkers Consortium - ABC-CT	EEG, eye-tracking, and related objective measures for stratification and clinical trial enrichment.	Autism spectrum disorder
EU-AIMS / AIMS-2-TRIALS	Multimodal stratification and outcome biomarkers spanning imaging, cognition, development, and immune biology.	Autism spectrum disorder
AMP Schizophrenia (AMP SCZ)	Multimodal longitudinal biomarkers and prediction tools for psychosis risk, clinical trajectories, and early intervention studies.	Schizophrenia / clinical high risk for psychosis
FNIH Biomarkers Consortium - Inflammatory Markers Project	Immune and inflammatory multicomponent biosignatures for early detection, subtyping, and treatment matching.	Alzheimer’s disease; major depressive disorder
AMP Alzheimer’s Disease (AMP AD)	Multi-omic target and biomarker discovery for molecular subtyping, precision medicine, and trial enrichment.	Alzheimer’s disease
Alzheimer’s Disease Neuroimaging Initiative (ADNI)	Imaging, fluid, genetic, and digital biomarkers for disease staging and trial-ready longitudinal cohorts.	Alzheimer’s disease
AMP Parkinson’s Disease (AMP PD)	Harmonized multi-omic, imaging, and clinical biomarkers for subtype identification and progression tracking.	Parkinson’s disease
Parkinson’s Progression Markers Initiative (PPMI)	Longitudinal clinical, imaging, fluid, genetic, and digital biomarkers for risk, onset, and progression.	Parkinson’s disease
AMP Amyotrophic Lateral Sclerosis (AMP ALS)	Fluid, molecular, digital, and clinical biomarkers together with clinical outcome tool development.	Amyotrophic lateral sclerosis
C-Path - CPAD	Integrated trial databases, biomarker-informed disease progression tools, and regulatory decision-support methods.	Alzheimer’s disease and related dementias
C-Path - CPP	Integrated databases, biomarker-enabled trial enrichment, digital tools, and progression modeling.	Parkinson’s disease

Examples are illustrative rather than exhaustive and emphasize multi-stakeholder pre-competitive programs that support biomarker discovery, validation, data harmonization, or regulatory-enabling tool development for CNS drug development. ABC-CT = Autism Biomarkers Consortium for Clinical Trials;

*ADNI* Alzheimer’s disease neuroimaging initiative, *AMP* accelerating medicines partnership, *C-Path* critical path institute, *CPAD* critical path for Alzheimer’s disease, *CPP* critical path for Parkinson’s, *FNIH* foundation for the national institutes of health, *PPMI* Parkinson’s progression markers initiative.

To realize this vision, a shift in research design and incentive structures is needed. Lessons from neuroscience, immunology, and oncology converge on the need for precompetitive, multimodal, and stakeholder-aligned strategies that unify academia, regulatory agencies, and industry. The scale and complexity of psychiatric biomarker discovery, spanning neuroimaging, fluid-based assays, behavioral phenotyping, and multivariate computational models, exceed what individual labs or companies can accomplish independently. Instead, what is required are open-science consortia with shared standards for data curation, biomarker qualification, and interoperability across platforms and studies.

Developing this infrastructure will not only accelerate progress toward mechanistically informed trials, but it will also lower the barrier for repurposing existing drugs and validating novel therapeutics. A neuroscience-guided, biomarker-driven approach to trial design rooted in dimensional phenotyping offers the best opportunity to overcome historical limitations and usher in a new era of targeted, effective psychiatric treatments.

This position paper addresses three central questions: (1) why biomarker development in psychiatry has lagged behind other therapeutic areas; (2) what scientific, regulatory, and structural barriers must be overcome; and (3) what coordinated actions across academia, industry, and regulatory agencies are required to establish clinically actionable biomarkers.

### Definitions and context of use

Biomarkers are defined as measurable indicators of normal biological processes, pathogenic mechanisms, or pharmacologic responses to therapeutic interventions [8]. These objective characteristics can be measured accurately and reproducibly, serving as critical tools in drug development, clinical practice, and regulatory decision-making. The FDA and NIH describe seven categories of biomarkers: (1) *risk/susceptibility* (i.e., identify likelihood of disease onset/progression), (*2) diagnostic*, (3) *prognostic*, (*4) monitoring* (i.e., repeated measures to assess the status of a condition or an exposure), (5) *predictive* (i.e., identifying those more likely to experience a certain result), (6) *pharmacodynamic/response* (i.e., demonstrates a biological response has occurred), and (7) *safety* (i.e., assesses for the presence or extent of toxicity). Within a regulatory framework, each biomarker must have a clearly defined *context of use* (COU)—a comprehensive statement describing its specific purpose and application in drug development and evaluation [9]. A COU is partially determined by which category of biomarker is described but also must incorporate how the biomarker will be used during drug development. For example, several types of biomarkers (e.g., risk, prognostic, predictive) could be used for *stratification* (i.e., to select/enrich subgroups) (Tables 3 and 4).﻿ There are three pathways to regulatory acceptance of biomarkers:

1. Scientific consensus based on published peer-reviewed scientific studies (e.g., hemoglobin A1c as a marker for blood sugar control in diabetes).
2. Validation of a biomarker in a drug development program under an investigational new drug application (IND)
3. A regulatory qualification program where a biomarker is accepted by regulators for use in drug development programs for a specific COU. A “qualified” biomarker has a reliable, specific interpretation and application for drug development—thereby ensuring clarity and consistency in its meaning [10]. Examples of pre-competitive efforts focused on biomarkers’ identification and validation are listed in Table 2.

**Table 3:** Contexts of use for biomarkers in psychiatric drug development.

Biomarker and Context of Use	Definition	Purpose/Example Use Case
Predictive Biomarker for Study Enrichment	Identify extent to which individuals may be likely to respond to a particular treatment	Used to personalize therapy selection and enhance efficacy signal detection in trials
Reasonably Likely Surrogate Biomarkers	Supported by strong mechanistic and/or epidemiologic rationale such that an effect on the surrogate is expected to be correlated with an endpoint intended to assess clinical benefit in trials [123]	May support approval when direct outcomes are impractical to assess in short-term trials. Requires postmarketing confirmatory trials; *Example: NfL as a reasonably likely surrogate for approval of* Qalsody *(tofersen) in SOD1ALS* [124]
Validated Surrogate Endpoints	Supported by a clear mechanistic rationale and clinical data providing strong evidence that an effect on the surrogate endpoint predicts specific clinical benefit	May enable traditional approval without the need for additional studies to demonstrate the clinical benefit directly; Surrogate biomarkers may also be valuable to manage patient burden; *Example: BMD as a surrogate endpoint for Osteoporosis Drug Development* [125]
Diagnostic Biomarker for Stratification	Select or enrich trial populations with shared biological characteristics [123]	Used in trial design to reduce heterogeneity in subgroups and examine differential response
Risk/Susceptibility Biomarkers	Identify likelihood of disease onset or progression	May be used in early detection, preventive interventions, or prognostic enrichment strategies

**Table 4:** Current guidance, gaps, and barriers for biomarkers in psychiatry.

Domain	Key Highlights	Implications for Psychiatry
Current Regulatory Guidance	- IND-stage biomarker inclusion is the primary pathway for biomarker integration in drug development, allowing flexible use throughout clinical trials	- Psychiatric drug development can leverage IND-stage biomarkers for mechanistic insights and early efficacy - BQP remains underutilized due to its time and resource demands (particularly by single academic centers)
	- FDA enrichment guidance supports prospective patient subgroup selection to enhance signal detection - BQP offers formal biomarker qualification for defined contexts of use, enabling cross-program acceptance, although it is not required	
Identified Gaps	- Gaps in mechanistic understanding of candidate biomarkers in psychiatry - Absence of successful psychiatric precedents (vs. AD or ALS in neurodegeneration) - Perceived lack of clear paths for composite/multimodal and AI-derived biomarkers - Limited clarity on reasonably likely surrogate endpoint utility and/or acceptance in psychiatry	- Precompetitive consortia are needed for better translation from discovery to implementation - Uncertainty around evidentiary standards and regulatory expectations hinders innovation in multimodal biomarker development - Limits the development of reasonably likely surrogate endpoints/biomarkers
Barriers to Translation	- BQP pathway is slow, expensive, and not feasible for many sponsors - IND route is faster but often engaged too late - AI-based methods risk overfitting; prospective validation is essential - Context of use often unclear; temporal stability under-addressed - Lack of standardization across sites/labs - Digital tools reveal new subgroups not mapped to DSM nosology	- Need for earlier integration of biomarkers in trials and clearer COU - Standardization and precompetitive frameworks (e.g., modeled after ADNI) are critical - Digital biomarkers challenge traditional classification systems

*AD* Alzheimer’s disease, *ADNI* Alzheimer’s disease neuroimaging initiative, *AI* artificial intelligence, *ALS* amyotrophic lateral sclerosis, *BQP* biomarker qualification program, *COU* context of use, *DSM* diagnostic and statistical manual of mental disorders, *FDA* food and drug administration, *IND* investigational new drug.

Because psychiatric disorders reflect complex interactions among genetic, molecular, neural, and environmental factors, a single biomarker is unlikely to capture the multidimensional biology underlying most conditions. Instead, integrated approaches may be needed to derive convergent evidence from multiple complementary measures. *Multicomponent biomarkers* combine two or more individual biomarkers—potentially within a single modality—into a composite index that provides greater predictive or diagnostic power than any single measure alone [8]. In contrast, *multimodal biomarkers* integrate data across distinct measurement modalities, such as molecular assays, neuroimaging, electrophysiological signals, and digital phenotyping, to represent different biological and behavioral levels of disease [11, 12]. These multimodal frameworks can better reflect the systems-level nature of psychiatric pathophysiology and support the development of biologically grounded, mechanistically informed therapeutic strategies.

In the context of drug development, biomarkers play several specialized roles that are particularly relevant to regulatory pathways. Biomarkers used for *enrichment* and *stratification* may be used to identify and select patient subpopulations who are more likely to share common biological or disease characteristics, thereby improving trial efficiency and enabling more precise therapeutic targeting. Biomarkers with this context of use may reduce heterogeneity in study populations and may enhance the ability to detect treatment effects. *Predictive biomarkers* may inform the likelihood of clinical response (and/or extent of response) from a particular therapeutic product or class, distinguishing patients who are more likely to respond favorably from those who may not benefit or could experience adverse effects. Unlike prognostic biomarkers that indicate disease outcome regardless of treatment, predictive biomarkers can directly guide treatment selection decisions.

*Surrogate* endpoints are biomarkers that are intended to substitute for a clinical endpoint and are expected to predict clinical benefit (or harm or lack of benefit) based on epidemiologic, therapeutic, pathophysiologic, or other scientific evidence. A validated surrogate endpoint is a surrogate endpoint that is supported by a clear mechanistic rationale and clinical data providing strong evidence that an effect on the surrogate endpoint predicts a specific clinical benefit. A validated surrogate endpoint can be used to support the traditional approval of a drug in a defined context without the need for additional studies to demonstrate the clinical benefit directly. In contrast, a reasonably likely surrogate endpoint (RLSE), while supported by strong mechanistic, therapeutic, pathophysiologic, or other evidence, does not meet the standard for a validated surrogate endpoint. RLSEs can support the use of the accelerated approval pathway for drugs intended to treat serious conditions that fill an unmet medical need. In these cases, accelerated approval may be granted when a drug is shown to have an effect on a surrogate endpoint or intermediate clinical endpoint that is reasonably likely to predict clinical benefit, with subsequent confirmatory studies required to verify the anticipated clinical benefit. FDA’s accelerated approval regulations specify that accelerated approval is available for drugs that provide a meaningful therapeutic benefit over existing treatments. Accelerated approval has been used in settings in which the disease course is long or the clinical outcome events intended to be reduced by the drug are infrequent. Surrogate endpoints or intermediate clinical endpoints have the potential to detect the drug effect that may predict clinical benefit earlier than endpoints showing clinical benefit [13].

Consequently, for many psychiatric disorders, validated clinical outcome assessments remain practical and accepted primary endpoints for evaluating treatment efficacy within conventional trial durations. As a result, the most immediate opportunities for biomarker development are likely to involve enrichment, stratification, target engagement, pharmacodynamic, safety, and early-response applications rather than surrogate endpoints. This should not be interpreted as diminishing the long-term importance of surrogate biomarkers. Rather, as biologically grounded signatures become more reproducible, mechanistically specific, and clinically validated, they may ultimately provide more objective and precise indicators of disease biology and treatment response than symptom-based measures alone. Realizing this vision will require biomarker development programs that are designed from the outset around clearly defined clinical decisions and regulatory contexts of use, while progressively incorporating considerations related to clinical workflow, scalability, interpretability, and implementation as biomarkers mature toward routine clinical deployment.

### Regulatory landscape and identified gaps

#### Current guidance

Current regulatory guidance highlights the use of biomarkers and enrichment strategies to improve trial efficiency and therapeutic precision. For instance, the *FDA Guidance for Industry on Enrichment Strategies* encourages the prospective selection of patient subgroups most likely to respond to treatment or progress in disease, thereby reducing heterogeneity and enhancing signal detection—an essential consideration in psychiatric drug development [14]. However, while enrichment strategies can increase trial efficiency and precision, they may have important implications for the population for which a product is ultimately indicated. The benefits of enrichment for improving signal detection should be weighed against considerations of generalizability to broader patient populations who may receive the treatment in clinical practice.

To streamline biomarker use in clinical research, the FDA has introduced several strategic initiatives to support biomarker use in drug development, recognizing their critical role in advancing precision medicine and improving clinical trial efficiency.

The most common and pragmatic pathway for biomarker integration in drug development is through direct inclusion at the development stage—within an *Investigational New Drug* (IND) application—or throughout the different phases of clinical development. This approach has proven highly flexible and widely used, representing the primary mechanism by which biomarkers gain regulatory endorsement in drug development. This route enables sponsors to incorporate exploratory biomarkers directly into clinical trials to support early efficacy signals, dose optimization, pharmacodynamic insights, and mechanistic hypotheses. While these biomarkers may not be formally qualified, their pragmatic use facilitates iterative learning that can later support clinical validation or qualification. This approach is increasingly aligned with broader regulatory trends favoring adaptive, data-driven trial designs in precision medicine that are relevant to psychiatry and neuroscience [15].

Alternatively, the Biomarker Qualification Program (BQP) offers a formal pathway for regulatory acceptance of biomarkers within a defined context of use [8] (Table 4). However, it is important to note that BQP is not a requirement for use of biomarkers in clinical trials.

#### Potential gaps

Despite the availability of multiple regulatory pathways for biomarker use as outlined, several critical perceived gaps remain that disproportionately affect psychiatric drug development (Table 4). Most notably, there are few well‑established psychiatric precedents in which biomarkers have achieved regulatory acceptance comparable to those seen in neurological disorders such as AD and ALS. This absence of precedent contributes to uncertainty among sponsors regarding the evidentiary standards required for successful regulatory engagement and limits confidence in biomarker‑driven development strategies.

In addition, there remains perceived ambiguity surrounding the regulatory treatment of composite and multimodal biomarkers, which integrate information across multiple biological and behavioral domains. While such approaches may be particularly well‑suited to capturing the multidimensional nature of psychiatric disorders, existing regulatory guidance has largely focused on single‑measure biomarkers, leaving unclear how evidence for combined or algorithm‑based markers should be generated, evaluated, and interpreted.

Finally, there is limited publicly available guidance specific to the acceptance of predictive biomarkers in psychiatry. Although general regulatory frameworks exist for reasonably likely surrogate endpoints (RLSE) and intermediate endpoints, their application to psychiatric indications has remained underdeveloped, in part due to the availability of direct clinical outcome measures and the absence of clearly defined pathophysiological anchors. Together, these gaps underscore the need for greater clarity, precedent‑setting examples, and structured dialogue among industry, sponsors, academic, and regulatory stakeholders to enable biomarker innovation in psychiatric drug development.

Importantly, the largest gap may be the intersection between academia and industry in translating neuroscience discovery. Based on experience with AD biomarkers, it is felt that, at least in neurodegenerative disorders, there are clear precedents to follow. Rather, to generate the needed quality data in psychiatric disorders for biomarker identification and validation, *precompetitive consortia* need to play an important and potentially critical role, given the limitations of what can be accomplished through grant mechanisms or competitive investments by companies.

#### Current barriers

The translation of biomarkers from discovery to application in psychiatric drug development continues to face critical obstacles that hinder progress toward precision medicine (Table 4). Among these are both the lack of necessary scientific understanding of pathology and persistent knowledge gaps regarding the most effective pathways for biomarker integration into clinical trials. While the formal biomarker qualification pathway administered through the BQP exists as an option, it is important to clarify that qualification is not required for biomarker use in drug development. Biomarkers can be, and routinely are, incorporated directly into clinical trials through IND applications without formal qualification. However, misconceptions about regulatory requirements may create perceived barriers that discourage biomarker adoption.

The BQP pathway, when pursued, remains time- and resource-intensive, often requiring years of development and substantial financial investment—resources that may exceed the capacity of individual sponsors, particularly in academia or smaller biotech sectors. These challenges are compounded by misalignment between academic researchers, industry sponsors, and regulators regarding optimal biomarker implementation strategies, underscoring the need for clearer psychiatric-specific guidance and more structured avenues for cross-sector collaboration to address both scientific gaps and misperceptions about regulatory pathways.

As noted above, the primary pathway for biomarker integration in psychiatric drug development is through direct inclusion within IND applications. This approach is favored by sponsors because it offers greater speed and flexibility, allows protection of intellectual property, and enables the inclusion of exploratory biomarkers in clinical trials without undergoing formal qualification. However, this strategy is frequently implemented too late in the development process, and is often lacking sufficient early-phase evidence to support meaningful regulatory decision-making. Moreover, early phase exploratory biomarkers have yet to prove useful in Phase 3 studies in psychiatry. Optimal use of the IND pathway requires earlier integration of biomarker strategies—ideally beginning in Phase 1—paired with transparent data sharing to accelerate downstream validation. Additional common challenges are the lack of analytical validation or standardization, which prevents exploratory biomarkers used in one program from being effectively translated to others, as well as the lack of naturalistic studies—or availability of phenotyped cohorts—for use as real world evidence comparators. Intellectual property concerns also represent a significant barrier to pre-competitive collaborative efforts that could advance biomarker development and validation across the field.

Methodological concerns present another layer of challenge, especially as *artificial intelligence* (AI) and machine learning techniques become increasingly central to biomarker discovery. Post hoc identification of biomarker-defined subgroups in negative trials carries a high risk of overfitting or generating spurious findings. While the FDA has issued guidance supporting the use of AI in drug development [16], it emphasizes the importance of prospective validation, particularly the inclusion and testing of biomarker-negative patients, to guard against misleading subgroup effects. A number of protective measures are available and recommended to safeguard the development of AI-derived and multimodal biomarkers, including locked models, prespecified analysis plans, external validation, calibration assessment, missing-data handling, sensitivity analyses across demographic groups, device-version tracking, and prospective testing in biomarker-positive and biomarker-negative participants. FDA’s 2025 AI guidance further recommends a risk-based credibility assessment framework that aligns model validation with the intended context of use, providing an important regulatory framework for AI-enabled biomarker development.

An additional conceptual barrier lies in the incomplete articulation of COU in many biomarker development programs. Frequently, identified subgroups lack defined therapeutic implications, or the biomarker’s utility in guiding trial design remains vague. Moreover, few programs explicitly address temporal stability—how a biomarker’s performance may shift due to illness progression, treatment effects, or normative developmental changes (e.g., in pediatric populations). While not all such challenges must be fully resolved prior to testing, a biomarker that demonstrates biological plausibility and early-stage robustness may still warrant development, provided uncertainties can be resolved with future data.

Technical issues in analytical standardization persist across platforms and laboratories. Many psychiatric biomarker initiatives lack common data elements (CDEs), reference assays, or harmonized protocols, impairing reproducibility. Lessons can be drawn from fields such as Alzheimer’s disease research, where the Alzheimer’s Disease Neuroimaging Initiative (ADNI) established unified biomarker standards that have accelerated clinical translation [17]. This highlights the importance of precompetitive consortia and data-sharing initiatives to de-risk early-stage biomarker development and build community consensus.

Analytical validation of a biomarker is also critical—it is a crucial process of proving that a test accurately and reliably measures a specific biological marker (like a protein or gene) by assessing its performance characteristics (sensitivity, specificity, precision, accuracy, etc.), to ensure consistent, high-quality data for intended clinical use. In general, this is not performed in patient samples and instead is often just an assessment of the tool itself. It does not necessarily inform on any clinical meaning. Instead, it establishes technical fitness, determining the test’s limits (detection, quantitation) and reproducibility, which is essential before linking the biomarker to actual patient health outcomes. It is also distinct from preanalytical considerations, although some companies/manufactures do include preanalytical assessments as part of the analytical validation. Often biomarker tests—protein assays, NGS, genetic tests, etc.—are run in trials without the proper analytical validation. As a result, a conclusion on the clinical meaning is made, but the test was not appropriately developed/assessed to inform that clinical interpretation and cannot be moved forward.

Finally, the expanding use of digital phenotyping and other novel biomarker modalities introduces an additional new challenge: these methods often reveal population subtypes that do not map neatly onto existing diagnostic categories, or do not map onto other biotypes, depending on the types of data included. As a result, demonstrating the clinical utility, prognostic relevance, and regulatory acceptability of these data-driven frameworks requires thoughtful validation strategies and could ultimately challenge the validity of traditional classification systems.

### Real-world case studies in biomarker development in psychiatry

Biomarker studies in psychiatry prior to 2010 played an important role in highlighting the necessity of rigorous standardization in data acquisition paradigms and analytic assays if replication was to be achievable. One notable success from this era was the development and application of PET ligands, particularly for targets such as dopamine D2 receptors, where different ligands could reliably yield comparable estimates of receptor occupancy across compounds [18]. Importantly, PET ligand development could be achieved within individual academic or industry laboratories and subsequently disseminated to the broader field, demonstrating that large-scale collaboration was not required for ligand discovery per se [19]. However, broader validation of PET biomarkers, particularly for uses extending beyond direct pharmacologic assessments of target engagement, has generally required larger, coordinated efforts [20, 21].

These early PET studies illustrate both the strengths and limitations of target engagement biomarkers in psychiatry. While receptor occupancy measures provided clear evidence of central nervous system penetration and pharmacodynamic effects, they did not address critical downstream questions related to patient selection, treatment response prediction, or clinical enrichment. As such, even well-validated pharmacologic biomarkers did not, on their own, resolve the broader challenges of heterogeneity and variable clinical response that characterize psychiatric disorders.

Since 2010, a limited number of studies—many supported by the National Institute of Mental Health (NIMH)—have sought to more explicitly link measures of target engagement, functional brain changes, and clinical outcomes [22]. These efforts have employed a range of experimental approaches, including biochemical measures such as receptor occupancy or CSF analytes, alongside functional readouts derived from functional magnetic resonance imaging (fMRI) or pharmaco-EEG. In several cases, these biological measures were related to changes in subjective symptom ratings or performance-based outcomes, such as cognitive measures. Notably, however, these studies have not generally progressed to using biomarkers prospectively to select or enrich patient populations in interventional clinical trials.

Taken together, these examples underscore a recurring pattern in psychiatric biomarker research: demonstration of biological effects in the brain has not yet fully translated into biomarker-guided trial design or regulatory decision-making. This gap does not reflect a failure of individual biomarkers, but rather the absence of validated frameworks for moving from mechanistic measurement to clinical stratification. These experiences highlight the need for *coordinated, precompetitive strategies* that extend beyond demonstration of target engagement to address reproducibility, context of use, and scalability—issues that are central to regulatory acceptance and clinical utility.

Below we focus on several consortia designed for exploring biomarkers that can be applied to either enrich populations or predict response to a specific class of drugs. The following case studies which have generated results each highlight different issues:

#### The EMBARC study

The *Establishing Moderators and Biosignatures of Antidepressant Response in Clinical Care* (EMBARC) study was an NIMH-funded, multisite, double-blind, randomized clinical trial designed to identify clinical and biological predictors (moderators) and early indicators (mediators) of antidepressant treatment response in MDD. EMBARC enrolled several hundred outpatients with early-onset, recurrent, non-psychotic MDD and randomized participants to an 8-week course of sertraline or placebo [23]. Non-responders to either treatment were subsequently switched in a second treatment phase to either bupropion (an antidepressant with a distinct mechanism of action from sertraline) for sertraline non-responders or sertraline for placebo non-responders. A defining feature of EMBARC was the systematic, prospective collection of a broad range of candidate biomarkers, including clinical phenotypes, multimodal neuroimaging (task-based and resting-state fMRI, DTI, arterial spin labeling), electrophysiology (EEG), cognitive and behavioral measures, and blood-based biomarkers, acquired using standardized protocols across sites. The long-term objective was to integrate these multimodal data into a composite Depression Treatment Response Index (DTRI) capable of guiding individualized antidepressant selection.

Analyses emerging from the EMBARC dataset have suggested that combinations of neurobiological and clinical features might modestly but meaningfully predict antidepressant and placebo response. For example, pretreatment EEG measures, including theta activity, in combination with clinical variables, have been shown to predict remission and placebo response with moderate accuracy [24]. Similarly, functional neuroimaging measures related to reward processing and frontostriatal circuitry have been implicated as moderators of sertraline response [25, 26]. These findings underscore the relevance of neural circuit-based markers, particularly those related to reward and cognitive control systems, in understanding heterogeneity in antidepressant outcomes and support the concept that depression subtypes defined by neurobiological profiles may have differential treatment sensitivity [27].

The EMBARC study provides a potential framework for biomarker development and validation in psychiatry by embedding comprehensive multimodal assessment within a rigorously controlled clinical trial. Rather than relying on single biomarkers, EMBARC operationalized the concept of biosignatures—integrated sets of markers spanning clinical, behavioral, and biological domains—to better capture the complexity of MDD. The study’s emphasis on protocol harmonization, centralized quality control, predefined analytic strategies, and public data sharing directly addresses longstanding barriers to reproducibility and generalizability in psychiatric biomarker research. The availability of EMBARC data has further enabled secondary analyses using machine learning and multivariate modeling approaches, facilitating independent validation efforts and hypothesis generation across the field. Lessons learned from EMBARC highlight several critical considerations. First, *it is important to consider multimodal integration*, as no single biological measure is likely to achieve sufficient predictive validity in isolation; however, such integration also limits statistical power. Second, the *sample size* may be a more limiting factor rather than the modality of included biomarkers, particularly when sample size is small. Third, *standardization of acquisition and analysis across sites is necessary* to minimize noise and enhance reproducibility. Fourth, *longitudinal assessment*, particularly early changes following treatment initiation, may be especially informative as mediators of outcome and actionable decision points in clinical care. To reduce the likelihood of a null trial, these considerations would emphasize a need to (1) collect far larger (standardized) samples, and (2) protect from false discovery with built-in, held-out data for validation, particularly when the disorder itself is not rare or associated with recruitment difficulty. Finally, EMBARC demonstrates the value of open science practices, with shared datasets accelerating discovery, replication, and refinement of candidate biomarkers.

#### ABC-CT and the FNIH ASD consortia

The *Autism Biomarkers Consortium for Clinical Trials* (ABC-CT) is a large, multi-site, longitudinal research initiative established to accelerate the development of objective, reliable biomarkers to support clinical trials and translational research in autism spectrum disorder (ASD), a highly heterogeneous neurodevelopmental condition. Funded through the Foundation for the National Institutes of Health (FNIH) Biomarkers Consortium [28] in partnership with NIMH, FDA, academia, and industry, ABC-CT systematically evaluated a set of candidate neurophysiological and neurobehavioral biomarkers, including EEG, eye-tracking (ET), and structured behavioral paradigms, in a deeply phenotyped cohort of children with ASD and typically developing controls [29]. A central goal of the consortium was to assess the feasibility, reliability, construct validity, and sensitivity to clinically meaningful differences of these measures under standardized, multi-site conditions approximating those of clinical trials. Notably, ABC-CT achieved an important milestone in psychiatric biomarker research when the EEG N170 face-processing measure and an eye-tracking social attention measure were accepted into the FDA’s Biomarker Qualification Program, marking the first psychiatric biomarkers to formally enter this regulatory pathway and establishing a precedent for objective stratification and enrichment tools in ASD trials [30]. Importantly, entry into the program should not be interpreted as formal biomarker qualification. Rather, it represents an early milestone within a multi-stage regulatory process that includes subsequent evidentiary review before qualification can be achieved. Nevertheless, ABC-CT established an important regulatory precedent by demonstrating that psychiatric biomarkers can successfully enter this pathway.

Results from the ABC-CT demonstrate that select biomarkers exhibit psychometric properties suitable for use in interventional studies, while others highlight important limitations that inform future development. Eye-tracking measures of attention to faces and social scenes showed high acquisition success, strong short-term test-retest reliability across a six-week interval, and meaningful associations with clinical features such as verbal ability and autism symptom severity [29]. Similarly, EEG measures indexing face-sensitive neural responses, including the N170 event-related potential, demonstrated stability and group-level discrimination, supporting their potential role as stratification biomarkers rather than direct outcome measures [30]. The ABC-CT provides a compelling model for biomarker development in psychiatry by integrating rigorous methodology, longitudinal assessment, and early regulatory engagement. Several key lessons emerge from this work. As with EMBARC, *multimodal approaches* for biomarker development to capture heterogeneity, and harmonization of protocols were critical. Furthermore, *assessing biomarker stability and sensitivity to change* over clinically relevant timeframes is necessary to define context of use, whether for stratification, enrichment, or outcome measurement. Additionally, ABC-CT highlights the *value of open science and early regulatory engagement*, demonstrating that psychiatric biomarkers can successfully enter formal regulatory qualification pathways when developed within structured, collaborative frameworks.

#### EU-AIMS consortium

The *European Autism Interventions – A Multicentre Study for Developing New Medications* (EU-AIMS) consortium, funded through the Innovative Medicines Initiative, represents one of the first large-scale European efforts to identify and validate objective biomarkers for ASD by integrating basic science, clinical characterization, and translational research across institutions and disciplines. Similar to ABC-CT, EU-AIMS was established to address the profound heterogeneity of ASD by assembling extensive longitudinal cohorts, including the Longitudinal European Autism Project (LEAP), which enrolled hundreds of children and adults with and without ASD and comprehensively characterized them across clinical, neurocognitive, neuroimaging, biochemical, and genetic domains with standardized protocols to identify stratification biomarkers and inform subgroup-specific hypotheses of pathophysiology and treatment sensitivity. LEAP’s design exemplifies a precision-medicine paradigm by moving beyond traditional case-control comparisons to focus on within-group variability and the validation of stratification markers that delineate biologically and clinically meaningful ASD subtypes—a crucial step toward personalized intervention strategies in psychiatry [31]. The EU-AIMS framework also pioneered collaborative networks and methodological harmonization that enabled regulatory engagement and encouraged subsequent initiatives such as the AIMS-2-TRIALS, thereby illustrating how structured, multi-site observational research with longitudinal follow-up, comprehensive phenotyping, and cross-domain biomarker assessment can advance the field toward robust, generalizable biomarkers with translational relevance for clinical trials and individualized care in ASD and potentially other neuropsychiatric conditions [32, 33].

Of note, AIMS2-TRIAL established a network following the biomarker discovery phase aimed at investigating and repurposing compounds in interventional proof-of-concept trials. This pre-competitive research consortium model, which is also shared by the *Accelerating Medicines Partnership® Schizophrenia* (AMP SCZ), allows promising biomarkers to be investigated longitudinally in response to treatment.

#### AMP-SCZ

The AMP-SCZ program is a large-scale, global consortium established in 2020 to catalyze the development of biomarkers and analytic tools that can predict clinical trajectories and treatment outcomes in individuals at clinical high risk (CHR) for psychosis and ultimately accelerate the development of more effective interventions for schizophrenia and related disorders. AMP-SCZ operates as a public-private partnership involving the National Institute of Mental Health (NIMH), FDA, the European Medicines Agency (EMA), academic institutions, industry, and advocacy organizations, with harmonized protocols implemented across more than 40 international sites to collect deeply phenotyped longitudinal data on approximately 1977 CHR participants and 640 matched controls over a 2-year period [34, 35].

The study integrates multimodal biomarker domains—including neuroimaging, electrophysiology, fluid biospecimens, cognition, digital phenotyping, speech and facial expression measures, genetic and environmental data—to develop and validate multivariate prediction algorithms that differentiate individuals destined for psychosis conversion from those with remission or persistent subthreshold symptoms, as well as to identify early indicators of treatment response [34, 35].

The value of the ongoing observational study is in its scale, harmonization, and longitudinal multimodal biomarker, cognitive, digital, and clinical assessments. The recently initiated Proof of Principle study is assessing these measurement tools for their utility in early stage clinical trials in the CHR population [36, 37]. Nonetheless, by uniting large-sample prospective cohort design, standardized data acquisition and quality control, and open-science data sharing through repositories such as the NIMH Data Archive, AMP-SCZ exemplifies a modern, collaborative approach to psychiatric biomarker development that enhances reproducibility and hopes to have utility for stratification, enrichment, but also in predicting clinical endpoints, and monitoring change in clinical symptoms over time and, ultimately, personalized preventative strategies for psychosis.

### Candidate biomarker modalities and examples

In the absence of clearly defined pathophysiological markers (as seen in Alzheimer’s disease where PET ligands quantify amyloid or tau), psychiatric disorders lack equivalent targets, underscoring the need for novel, objective biomarker modalities. These should ideally transcend traditional symptom scales and self-report measures, which often fail to distinguish mechanistically distinct subgroups within heterogeneous diagnostic categories.

To meaningfully impact clinical practice and support drug development, biomarkers must be scalable, minimally burdensome, analytically rigorous, and operationalizable across trial contexts. They should demonstrate reliability, biological plausibility, and regulatory-relevant contexts of use, particularly where measures may act reasonably likely surrogates or intermediate endpoints. Increasingly, composite or multimodal panels, often integrating orthogonal data streams, are likely required to capture the complexity of psychiatric pathophysiology. *Artificial intelligence* (AI) and *big data platforms* may play a key role in discovering and validating such markers [38], though concerns around overfitting necessitate robust prospective validation.

The following classes of biomarker modalities may be among the most promising:

- **Fluid-Based and Genomic Biomarkers:**
  Including cytokines, kynurenine metabolites, brain derived neurotrophic factor, complement factors, and other proteomic analytes. Emerging proteomics platforms (e.g., Olink, SomaScan, NULISA) enable high-throughput, panel-based quantification to identify exploratory or potential biomarker candidates. Specificity remains a challenge, and integrating genetic data may help adjust for interindividual variation in proteomic expression, particularly given recent evidence of shared genetic overlap across common disorder categories (e.g., externalizing vs. internalizing) [39]. Genetic risk studies are relatively mature and inexpensive; however, common variants provide such low risk prediction that they are less likely to be useful, and PRS’s, while more robust [40, 41], are still relatively low in level of risk prediction and may have other limitations [42]. Other exploratory approaches include extracellular vesicles [43], transcriptomics [44, 45], and epigenetics [46], though reproducibility, scalability and clinical interpretability are still evolving [47].
- **Immuno-Metabolic Biomarkers:**
  Markers reflecting inflammatory, immune, and metabolic processes (e.g., cytokine profiles, acute‑phase reactants, lipid and glucose‑related measures) are attractive due to assay availability and scalability but currently face challenges in psychiatric applications related to limited disease specificity [48] and susceptibility to systemic confounds (e.g., comorbidity, lifestyle, medication effects) [49]. Their greatest potential may lie in stratification or predictive contexts of use, particularly when refined into multivariate signatures or integrated with CNS‑proximal measures to enhance interpretability and clinical relevance.
- **Digital Biomarkers:**
  Passive and active digital phenotyping from smartphones, wearables, and sensors, including actigraphy, heart rate variability, skin temperature, facial expression, eye gaze, speech and/or voice patterns, and gait dynamics. While promising for real-world measurement and scalability, linking digital signals to clinically meaningful outcomes can be challenging [50, 51]. Strategies aligned with FDA’s PFDD (Patient-Focused Drug Development) guidance may help advance the evidentiary framework [52].
- **Behavioral and Symptomatic Assessments:**
  It is important to distinguish that behavioral and cognitive assessments are not biomarkers but rather clinical outcomes or endpoints. Digital versions of traditional clinical assessments (e.g., increasingly validated ecological momentary assessment tools and cognitive tasks) and new test batteries offer scalable options for measuring behavioral and symptomatic outcomes. However, performance-related outcomes (whether digital or paper-based) should also demonstrate relevance to disease-specific functional measures and show particular value when validated in real-world settings [53, 54].
  In specific contexts, such as prodromal or clinical high-risk states for psychosis, attenuated symptoms and neurocognitive measures may warrant further investigation as potential predictive markers of disease trajectory. Ongoing efforts such as the AMP-SCZ consortium [ref] are systematically evaluating whether baseline cognitive and symptomatic assessments can predict conversion to psychosis or other clinically meaningful outcomes, which could inform their future use as prognostic or stratification tools in early intervention trials.
- **Cognitive Assessments:**
  Standard neurocognitive batteries are typically employed as clinical endpoints rather than biomarkers. While they may serve as anchors for functional impairment, though more work is needed to tie them to functional improvement, and despite the need for judicious selection of disorder-targeted tools [55, 56]. In some cases, incorporating cognitive measures into multimodal assessment panels alongside biological biomarkers may increase specificity and interpretability, particularly when aligned with mechanistic targets and used to characterize treatment-responsive subgroups.
  Notably, the MATRICS Consensus Cognitive Battery (MCCB) is a cognitive testing battery developed by a consortium effort in collaboration with FDA [57]. The FDA considers this battery validated for use as a clinical endpoints in clinical trials of cognitive impairment associated with schizophrenia (CIAS). The Measurement and Treatment Research to Improve Cognition in Schizophrenia (MATRICS) initiative in the early 2000s established consensus on methodology for treatment studies, including assessment strategies and trial designs for CIAS.
- **Electrophysiological and Network-Based Measures:**
  EEG, Magnetoencephalography (MEG), stereo-EEG, and deep brain stimulation (DBS)-derived signals enable assessment of large-scale brain networks and event-related potentials (e.g., mismatch negativity, P300, REM suppression) [58, 59]. EEG, and to some degree MEG, are scalable, pharmacologically sensitive, and well-suited to testing temporal dynamics. Advances in dry electrode technologies might further increase feasibility for clinical translation [60].
- **Structural and Functional Magnetic Resonance Imaging (MRI):**
  MRI-based modalities (including structural MRI, resting-state MRI and task-based fMRI, arterial spin labeling, and related network approaches) are especially informative in early-phase and mechanistic studies, where they can provide pharmacodynamic evidence of central nervous system engagement and support biologically informed stratification frameworks. Importantly, the reliability and translational utility of MRI measures are highly dependent on the intended context of use, analytic strategy, and study design. While some task-based whole-brain measures demonstrate only modest between-subject reliability, region-of-interest analyses, harmonized acquisition protocols, resting-state connectivity, cerebral blood flow measures, and within-subject designs may show substantially stronger reproducibility and translational utility [61–63]. Emerging precision neuroimaging applications further demonstrate the potential of biologically informed circuit-based biomarkers for patient stratification and treatment targeting [64, 65]. As with other biomarker modalities, successful implementation will require continued attention to standardization, multisite harmonization, and clearly defined contexts of use.
- **Positron Emission Tomography (PET) Imaging:**
  While resource-intensive, PET provides gold-standard quantification of target engagement, drug biodistribution, and synaptic plasticity markers (e.g., SV2A). Its use is best justified where pathophysiological targets are well-defined and is most common in proof-of-concept or mechanistic studies.
- **Utilizing and integrating biomarkers:**
- **Real-World and Longitudinal Sampling Platforms:**
  Increasing deployment of digital tools, ambulatory EEG [66], and home-based sample collection and assessments [67] enable longitudinal data capture ‘in the wild’ with low participant burden. In parallel, single-shot biological readouts (e.g., hair and teeth for stress biomarkers) offer temporally extended insights, without the need for repeated sampling [68, 69].
- **Multimodal Integration:**
  Approaches that combine two or more of the above domains are gaining traction, particularly those using integrated metric spaces (e.g., Riemannian embeddings) to harmonize disparate data types [70–74]. These may ultimately yield the most robust and generalizable biomarkers, especially when validated across precompetitive consortia. Importantly, multimodal platforms should be viewed primarily as discovery and validation frameworks; successful clinical implementation may ultimately depend on simplifying these approaches into scalable, interpretable, and operationally deployable biomarkers appropriate for their intended context of use.
- **Equity and Regulatory Considerations in Biomarker Discovery and Utilization:**
- Clinical and biomarker validation trials historically, and often by necessity, enroll a small subset of a given population in order to ensure robust and interpretable data outcomes. Much of the data collected about the efficacy of a novel pharmacological treatment outside the core clinical trials comes from real world studies or post-approval studies. Given the heterogeneity in psychiatric populations of aspects such as ancestry, age, sex, hormonal state, language, culture, socioeconomic status, comorbid physical illness, medication exposure, substance use, trauma history, sleep, diet, and trial site effects, it is crucial that biomarker development includes diversity targets, subgroup performance reporting, and validation in pragmatic and real world settings [75].
- Notably, digital biomarkers raise additional ethical, operational, and regulatory considerations beyond those encountered with many traditional biomarker modalities. These include protection of privacy, surveillance risk, informed consent, data ownership, algorithmic bias, firmware and device drift, participant adherence, missing data, cybersecurity, and equitable access to smartphones, wearables, and other digital technologies. Addressing these challenges will be essential to ensure that digital biomarkers are trustworthy, reproducible, and broadly deployable across heterogeneous healthcare settings.

#### Animal models and nonclinical approaches

Animal models remain critical for both biomarker discovery and mechanistic validation, particularly where preclinical work can inform translatable biological and pharmacodynamic markers. Preclinical and animal model systems play an important enabling role in biomarker identification and mechanistic understanding for precision psychiatry by providing experimental control that is not feasible in human studies. These systems allow investigators to directly link molecular, circuit-level, physiological, and behavioral measures under well-defined perturbations, thereby establishing causal relationships between candidate biomarkers and underlying pathophysiology. Importantly, animal models enable cross-level alignment of biomarkers—such as transcriptomic or proteomic signatures, electrophysiological readouts, and neurocircuit dynamics—with stress exposure, genetic risk, or pharmacologic manipulation, supporting construct and biological validity rather than symptom-only correlations [76–78]. When designed with translational intent, preclinical studies can prioritize biomarkers that are measurable across species (e.g., EEG oscillatory features, stress hormone dynamics, inflammatory markers), increasing the likelihood that findings will generalize to human clinical populations [79].

An important challenge with animal models is ensuring the *translational relevance* of the signs and symptoms that are operationalized by the model in the context of specific psychiatric conditions [78]. Historically, there have been transformational advances in understanding the brain mechanisms of stress and stress-related psychopathology by simply segregating animals into so-called “susceptible” and “resilient” subgroups, followed by comparisons across multiple domains (e.g., behavioral, physiological, and/or cellular endpoints) [80, 81]. The advent of machine-learning and AI may enable a greater focus on leveraging individual differences that can sometimes be obscured with dichotomous group assignments. Increased utilization of novel statistical approaches such as linear mixed models (LMMs) that utilize disaggregated data across numerous variables while ensuring that each individual’s data remains nested (i.e., linked to that individual) will unearth novel (but meaningful) correlations among endpoints that are far too complex to identify with the human eye. Combined with the increased use of strategies that are already well-established in humans—including the continuous measurement (i.e., over sustain periods of time) of objective (i.e., non-subjective) measurements of endpoints that can be derived from digital devices (e.g., activity, sleep, heart rate variability, blood pressure, gait metrics) [82]—future work in animal models may move toward embracing individual variability rather than devised approaches to minimize it. As an early example, recent work has established correlations between stress-induced changes in sleep architecture and anhedonia across individual mice that were not evident with dichotomous binning [83]. Application of these approaches across species represents an opportunity to improve alignment between psychiatry and neuroscience.

Beyond validation, nonclinical, or preclinical, systems are uniquely positioned to advance mechanistic understanding and inform biomarker-guided intervention development. Animal models allow for experimental dissection of circuits and cell types driving biomarker signals using modern tools such as optogenetics, chemogenetics, and cell-type–specific omics, clarifying whether biomarkers index vulnerability, disease state, compensatory processes, or treatment engagement [84–86]. These approaches support iterative, bidirectional translation, in which human-derived biomarkers guide hypothesis-driven animal experiments, and mechanistic insights from preclinical work refine biomarker selection and interpretation in clinical trials. Integrating preclinical validation into biomarker pipelines can therefore reduce false discovery, improve interpretability, and accelerate regulatory confidence by grounding candidate biomarkers in reproducible biological mechanisms rather than purely statistical associations. Regardless, animal models occupy an important niche in brain research and drug development, and ensuring their ability to predict outcomes in humans is an important goal that will require more deliberate focus as failures to translate accumulate [87, 88] and attitudes about the use of research animals shift [89].

### De-risking strategies for industry adoption

Successful translation of biomarker research into drug development pipelines requires a *multi-pronged de-risking strategy* to address *scientific*, *regulatory*, and *commercial* challenges. Below we outline each domain, with a focus on approaches that enhance reliability, reproducibility, and scalability.

#### Scientific De-risking

Biomarker discovery and validation in psychiatry face unique obstacles, including diagnostic heterogeneity and a paucity of clear pathophysiological anchors. To mitigate scientific risk, standardized platforms and harmonized trial protocols are essential [90], ideally supported through pre-competitive, non-proprietary sharing of protocols, techniques, and datasets (e.g., EMBARC, iSPOT-D *(*International Study to Predict Optimized Treatment in Depression [91]*)*, AMP-SCZ, and NESDA (Netherlands Study of Depression and Anxiety) [92]). Such open science models accelerate reproducibility testing and stakeholder trust, as well as enable analysis of very large datasets. Currently, data sharing activities are limited, and very frequently do not involve properly documented raw data or expeditious data acquisition periods, even though addressing such issues has been required by NIH for years. Finally, in addition to standardized protocols, scientific derisking requires inclusion of exploratory biomarkers in early phase clinical trials, as well as open data sharing, and dissemination of results in peer-reviewed publications. Thus, a culture shift is needed among academic and industry stakeholders around data inclusion, precompetitive planning and discussions, and data sharing.

Trial design is also critical. *Within-subject studies* (e.g., crossover trials like ketamine vs. placebo) and *short-term, parallel arm, applications* (e.g., early-phase proof-of-concept trials performed under double-blind, placebo-controlled conditions) are more efficient in revealing clinical efficacy signals and distinguishing them from nonspecific effects, while also allowing for individualized tracking of biomarker response. However, interpretation of crossover trial results may be difficult because of carryover effects, warranting appropriate design modifications (e.g., washout effects, placebo run-ins, run-outs, etc.). Relying on group-level comparisons of biomarker measures obtained at a single timepoint can obscure mechanistic effects, particularly where signal-to-noise ratios are low. A shift away from assumptions of direct causality between target engagement and symptom change is also warranted; indirect, cascade-based effects in complex CNS systems must be considered. Finally, targeted investment in safety and toxicology assessments—particularly for novel CNS mechanisms—will strengthen translational readiness and enable broader community learning through data sharing.

#### Regulatory De-risking

*Clarifying regulatory expectations early in development* is essential to avoid late-stage failures. This includes understanding the FDA’s requirements for biomarker-based enrichment strategies, acceptable entry points for IND submissions, and frameworks for post hoc analyses [9]. Establishing a robust evidentiary foundation for transdiagnostic constructs, supported by strong clinical proof points and aligned with regulatory guidance, will be key to gaining acceptance for nontraditional or multimodal/composite biomarkers.

Importantly, biomarker validation is a scientific necessity as much as a regulatory one. The FDA emphasizes that validation should demonstrate reliability, reproducibility, and relevance to clinical outcomes in a defined COU). This involves both *analytical validation* (ensuring consistent measurement performance across pre-analytical variables such as sample handling and storage, as well as assessment and understanding of the biomarker tests performance for the specific COU and *clinical validation* (demonstrating correlation with biologically meaningful and clinically actionable endpoints) [93]. Cross-cohort replication is essential to mitigate overfitting—particularly in psychiatric conditions characterized by biological heterogeneity.

Validation is distinct from *qualification*, which is a formal regulatory pathway enabling a biomarker’s use across multiple programs under a shared COU. Although qualification may support broader industry uptake, it is not a requirement for biomarker use in interventional trials. Modern validation frameworks are evolving to accommodate complex, multicomponent, and digital biomarkers, which require rigorous assessment of data integration methods and interaction effects across components.

#### Commercial De-risking

From a commercial standpoint, *scalability and cost-effectiveness* are paramount. Biomarkers that are low-cost, minimally burdensome, and easy to deploy (e.g., digital or fluid-based) are more likely to be adopted in Phase 2 and 3 trials and eventually in the clinical setting. These scalable tools should ideally be anchored by deeper, multimodal analyses at earlier stages, which establish mechanistic relevance and bridge to simplified markers at later phases.

Sponsors must also demonstrate trial efficiencies—fewer participants, stronger signal detection, and more efficient site activation. Moreover, linking biomarkers to differentiated efficacy and safety profiles supports downstream payer confidence. Ultimately, *de-risking strategies must extend beyond scientific robustness* to ensure that products supported by novel biomarkers meet the evidentiary demands of both regulators and healthcare systems. Data showing the robustness and replicability of clinical decision-making enabled by a novel biomarker, as well as its ability to demonstrate measurable clinical and functional improvement, is critical for support from regulatory agencies, payers, and healthcare systems. Notably, a move toward precision psychiatry can thereby enable a greater focus on value-based care models.

### Trial design frameworks

Advancing precision biomarkers in psychiatry requires trial designs that move beyond discovery toward *systematic validation*, *generalizability*, and *regulatory relevance*. Building on lessons from large consortia and mechanistic studies, complementary frameworks have emerged to support biomarker development across the translational pipeline.

#### Prospective, mechanism-engaged studies (fast-acting or experimental probes)

Prospective experimental designs that leverage *mechanistically targeted, fast-acting interventions* provide a powerful framework for identifying biomarkers that are proximal to pathophysiology rather than downstream clinical change. Pilot crossover or parallel-group studies using single-dose or short-course interventions allow investigators to tightly couple biological readouts to rapid symptom or circuit-level changes—examples include cross over trials of ketamine in depression, or short trials in psychosis-at-risk populations. Such approaches will like increase signal-to-noise and reduce confounds associated with chronic treatment exposure [94, 95]. However, crossover designs can be difficult to interpret when symptomatic improvement does not reliably reverse after initial response, as is often observed with rapid‑acting antidepressants; in such settings, treatment order effects, incomplete washout, and functional unblinding may confound subjective outcomes. Accordingly, the utility of these designs may depend less on speed of action per se than on the magnitude and durability of biological effects that can be measured independent of self‑report. These designs are particularly well-suited for discovering *state-sensitive* or *pharmacodynamic* biomarkers (e.g., EEG gamma power, functional connectivity shifts, or glutamatergic metabolites) that may serve as early indicators of target engagement or response prediction. When embedded within rigorous protocols that include baseline characterization, dense early sampling, and replication cohorts, such studies can generate candidate biomarkers that are subsequently tested in larger, later-phase trials, forming a translational bridge between neuroscience discovery and clinical development [76]. Notably, crossover paradigms may be better suited to evaluating biological or pharmacodynamic endpoints than to subjective symptom change, particularly when objective neural or molecular measures demonstrate reversibility even if clinical state does not.

#### Retrospective validation using existing large-scale trials and cohorts

Retrospective biomarker validation represents a complementary and highly efficient strategy, particularly when mining existing large clinical trials and observational cohorts that already include high-quality biomarker, imaging, or digital phenotyping data. Programs such as EMBARC, ABC-CT, EU-AIMS/LEAP *(European Autism Interventions and Longitudinal European Autism Project)*, and AMP-SCZ exemplify how deeply-phenotyped datasets enable post-hoc testing of candidate biomarkers across heterogeneous populations, treatment arms, and outcomes, supporting assessment of generalizability and robustness [23, 29, 31]. Retrospective analyses allow investigators to evaluate whether biomarkers identified in smaller mechanistic studies retain predictive or stratification value at scale, while advanced statistical and machine-learning approaches can integrate multimodal features into composite biosignatures. Critically, this framework emphasizes *validation rather than discovery*, helping to winnow large candidate sets to those most likely to succeed in prospective testing and regulatory qualification, provided that analytic plans account for multiplicity, overfitting, and cohort-specific biases [96].

#### Collaborative, standardized consortium models

Collaborative consortium-based models provide the infrastructure necessary to move psychiatric biomarkers from promising signals to clinically actionable tools. Modeled after successful efforts in neurodegenerative disease, such as ADNI and the Global Biomarker Standardization Consortium (GBSC), these frameworks emphasize harmonized acquisition protocols, centralized quality control, shared analytic pipelines, and early engagement with regulatory agencies to define *context of use* [17, 97–99]. In psychiatry, consortia such as ABC-CT and AMP-SCZ demonstrate how public–private partnerships can align academia, industry, regulators, and patient stakeholders around precompetitive goals, enabling large-scale replication, cross-site reliability testing, and regulatory-grade evidence generation. Key lessons from these models include the necessity of prespecified biomarker qualification plans, longitudinal designs matched to clinical trial endpoints, and open-science data sharing to accelerate independent validation. Together, collaborative consortia transform biomarker development from isolated discovery efforts into cumulative, field-wide enterprises capable of supporting precision medicine in psychiatry.

### Stakeholder incentives and translational levers

Progress in biomarker development for psychiatry requires *coordinated action across the translational ecosystem*. Stakeholders including pharmaceutical sponsors, digital and diagnostic technology developers, academic researchers, regulators, advocacy groups and payers, have aligned, yet distinct, incentives that can be leveraged to catalyze adoption. Central to this ecosystem is the meaningful involvement of people with lived experience and their care partners, whose perspectives are essential for defining clinically meaningful outcomes, acceptable benefit–risk trade‑offs, and real‑world feasibility [100].

Biopharmaceutical sponsors stand to gain from reduced development costs, accelerated timelines, and improved probability of technical success through earlier go/no-go decision-making. Biomarkers that enhance patient stratification or serve as pharmacodynamic indicators can substantially improve trial efficiency, especially in early-phase studies. *Shared use of precompetitive infrastructure*, including validation frameworks, data standards, and regulatory engagement strategies, enables industry actors to align around common evidentiary requirements while reducing redundant investment.

Diagnostic and digital health developers benefit from clarified regulatory pathways and clinical endpoints that are anchored in transdiagnostic constructs. As noted in earlier sections, biomarker validation is first and foremost a scientific endeavor. However, when embedded within coordinated qualification initiatives, it can de-risk downstream commercialization by aligning with well-defined *contexts of use* (COUs) accepted by regulatory agencies.

Regulatory agencies have a critical interest in harmonization across platforms and sponsors to streamline the review process, ensure patient safety, and increase public trust in novel endpoints. Consortia-based models that promote analytic transparency, real-world testing, and standardized data elements (e.g., CDISC standards) facilitate regulatory acceptance. Iterative discussions with regulators throughout the development process are essential to ensure alignment on data and methods. Additionally, sponsors may optionally pursue established FDA pathways such as the Drug Development Tool qualification, though these formal qualification processes are not required for biomarker use in clinical trials.

Payers and health systems may be most incentivized by outcome-based reimbursement models that use biomarker-informed patient segmentation to predict treatment response, reduce trial-and-error prescribing, and align with value-based care principles. In this context, demonstrating real-world utility and health-economic value of biomarkers (e.g., through adaptive trials or pragmatic registries) will be key. Scalable, low-cost biomarker platforms, especially those incorporating digital and fluid-based readouts, can facilitate broader deployment and coverage determinations.

Clinical investigators and academic consortia play a foundational role by validating biomarkers across diverse populations and settings, ensuring generalizability, and fostering the analytic rigor necessary to avoid overfitting. Their participation is also critical to establishing “translational levers” that link discovery-phase insights to regulatory and commercial endpoints. These include open-source repositories, multi-cohort data integration, and trial-ready infrastructure embedded within health systems or academic medical centers.

### Expanded regulatory landscape

The regulatory ecosystem for biomarker development is evolving rapidly, with agencies such as the FDA and European Medicines Agency (EMA) implementing new mechanisms to support both validation and qualification of innovative biomarker modalities. In the U.S., the FDA’s Innovative Science and Technology Approaches for New Drugs (ISTAND) [101] and Safer Technologies Program (STeP) [102] offer early engagement pathways designed to accelerate development and application of emerging tools in clinical trials, including digital biomarkers, wearable-derived signals, and fluid-based assays. The Critical Path Innovation Meetings (CPIM) framework [103] further facilitates collaborative, precompetitive dialogue between sponsors and regulators, particularly in areas not yet addressed by formal guidance. FDA’s digital-health-technology guidance covers remote data acquisition in clinical investigations and provides additional guidance, in particular related to wearables and smartphone-derived measures.

Despite these advances, few psychiatric biomarkers have moved beyond the exploratory phase. Common barriers include insufficient test–retest reliability, limited biological specificity, and lack of replication across diverse populations. To address this, early and iterative engagement with regulators is essential. Mechanisms such as mock submissions, parallel advice programs, and involvement of regulatory representation at meetings [1], can help clarify evidentiary requirements and improve translational viability.

Early and iterative engagement with regulators, including through pre-competitive collaborative initiatives, creates a crucial translational lever enabling earlier adoption of novel biomarker modalities, while encouraging shared scientific frameworks, regulatory harmonization, and innovation in underdeveloped domains such as psychiatry and neurology. When paired with proactive scientific de-risking, stakeholder alignment, and attention to commercial scalability , these mechanisms offer a clear pathway for next-generation biomarkers to impact patient care (see earlier sections on “Trial Design Frameworks” and “Stakeholder Incentives and Translational Levers”).

### Integration with ongoing initiatives

Multiple large-scale consortia and initiatives are already advancing key domains of biomarker science in psychiatry, offering natural alignment points with this roadmap proposed by the ACNP Precompetitive Stakeholder Task Force. Efforts such as the FNIH Biomarkers Consortium [28], the Accelerating Medicines Partnership for Schizophrenia (AMP-SCZ) [35], Remote Assessment of Disease and Relapse in Major Depressive Disorder (RADAR-MDD) [104], and the Coalition for Accelerating Standards and Therapies (C-Path/CFAST) [105, 106] collectively span a broad ecosystem—from multi-modal phenotyping and data standards to trial design innovation and regulatory science. Each program contributes distinct strengths that can be leveraged to accelerate biomarker identification, validation, and implementation across the development pipeline.

Integrating the present roadmap with these efforts ensures that definitions, evidentiary pathways, and methodological standards remain interoperable with global frameworks. Notably, this roadmap aligns with the goals of the *ECNP Precision Psychiatry Roadmap* [1], especially its emphasis on robust evidentiary thresholds, data harmonization, and operationalization of biomarkers into clinical development.

To minimize redundancy and amplify impact, the roadmap recommends *formal coordination mechanisms*: cross-consortium working groups, active liaison roles, and reciprocal participation in steering and technical committees. These mechanisms can help harmonize terminology, align evidentiary standards, and ensure methodological coherence. Shared use of clinical trial infrastructure, such as harmonized phenotyping batteries, CDEs, and compatible biofluid and neuroimaging pipelines, would reduce cost, increase interpretability, and foster reproducibility.

A particularly impactful opportunity lies in developing a federated but interoperable biomarker repository with a harmonized metadata schema. Such infrastructure would allow precompetitive pooling of longitudinal clinical, biological, and digital data, supporting validation across diverse populations and enhancing statistical power. Standardized metadata, including assay properties, device specifications, data quality indices, and clinical context of use, would promote transparency, facilitate regulatory review, and allow direct comparison of biomarker performance across platforms and populations. However, these goals will require proactive solutions to long-standing challenges associated with slow and inconsistent data sharing, even across major NIH-funded consortia.

By functioning as a connective hub among existing initiatives, the current ACNP precompetitive roadmap can help orchestrate a more cohesive, scalable, and globally relevant biomarker ecosystem in psychiatry—one that transitions reliably from exploratory signal to validated tool supporting both clinical decision-making and precise therapeutic innovation.

### Future challenges: bridging biomarker validation and clinical implementation

**Fig. 1:**
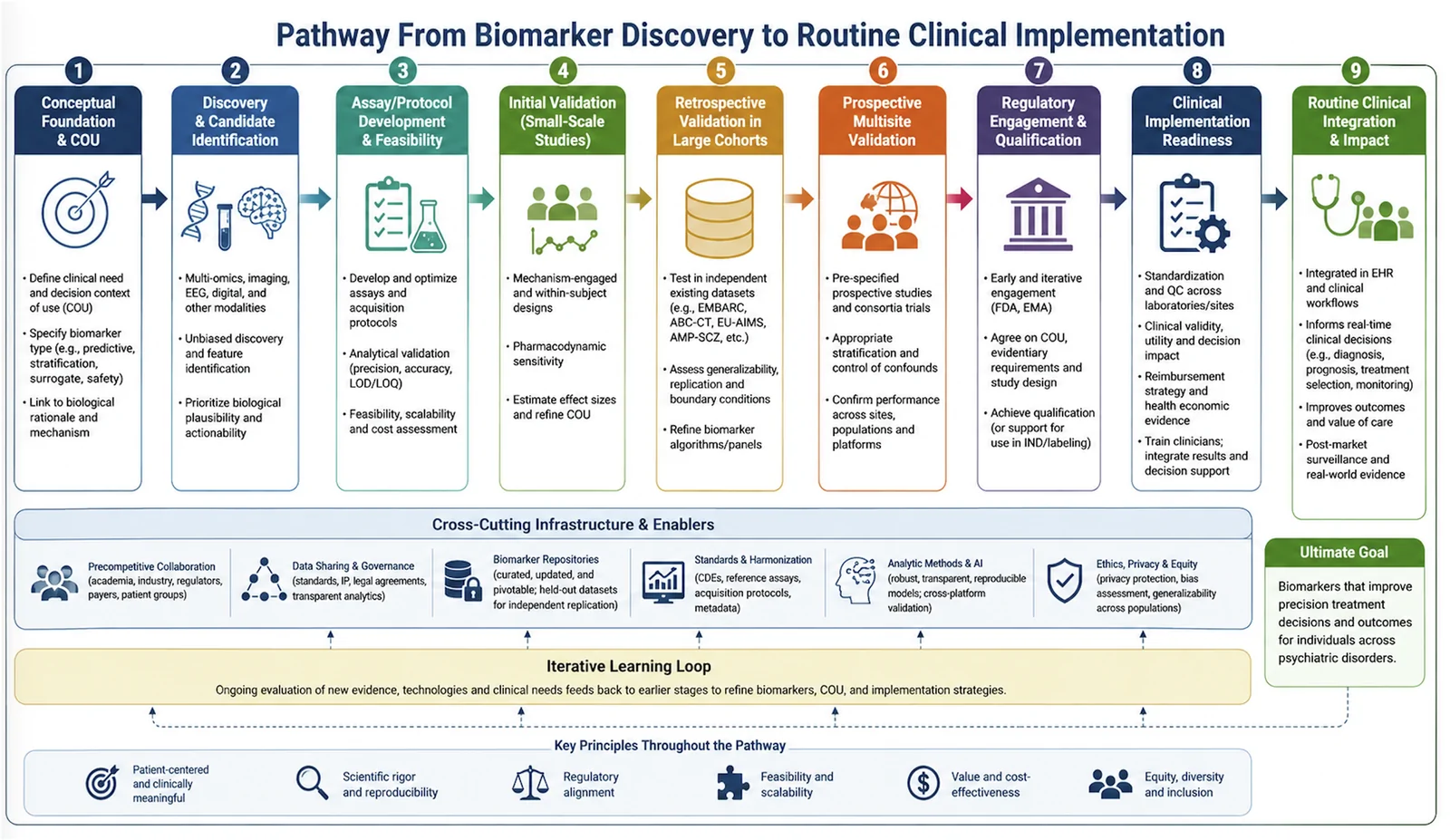
Pathway from biomarker discovery to routine clinical implementation. The framework depicts sequential stages of psychiatric biomarker development from defining clinical need and context of use through discovery, validation, regulatory engagement, and clinical integration. Cross-cutting precompetitive infrastructure and an iterative learning loop support reproducibility, scalability, regulatory alignment, and translation toward clinically meaningful use.

The roadmap presented here focuses primarily on the scientific, regulatory, and precompetitive processes required to identify and validate biomarkers suitable for psychiatric drug development (Fig. 1). However, successful validation does not guarantee routine clinical implementation. Moving biomarkers into clinical practice represents a distinct translational challenge that will require complementary advances in healthcare delivery, regulatory science, reimbursement, clinical workflow integration, and implementation research.

Future biomarker development programs may therefore benefit from considering operational deployment earlier in the translational pathway. Beyond analytical validity and regulatory qualification, biomarkers intended for routine use should ultimately demonstrate compatibility with existing healthcare infrastructure, support clear clinical decision-making, be scalable across heterogeneous practice settings, and generate evidence sufficient for payer adoption and reimbursement. These considerations are unlikely to determine early biomarker discovery but may increasingly shape biomarker selection and development as programs mature toward clinical implementation.

The operational questions surrounding clinical deployment, including workflow integration, data interoperability, interpretation, implementation across healthcare systems, and long-term sustainability, represent important areas for future multidisciplinary collaboration involving clinicians, health systems, regulators, industry, implementation scientists, and patient stakeholders. Addressing these challenges will be essential if validated biomarkers are ultimately to realize their potential in routine psychiatric care.

### Recommendations and next steps

Drawing on the challenges, case studies, and regulatory gaps detailed throughout this manuscript, the ACNP Precompetitive Stakeholder Task Force recommends the following coordinated actions to advance regulatory-ready biomarkers in psychiatry. These recommendations are designed to be mutually reinforcing—linking *conceptual clarity* (see sections “Definitions and Context of Use” and “Regulatory Landscape and Identified Gaps"), *empirical validation* (see sections "Real-World Case Studies”, “Candidate Biomarker Modalities”, “De-risking Strategies” and “Trial Design Frameworks”), and *translational infrastructure* (see sections “De-risking Strategies”, “Trial Design Frameworks”, “Stakeholder Incentives”, “Expanded Regulatory Landscape” and “Integration with Ongoing Initiatives”)—and to prioritize feasibility, scalability, and early impact within a precompetitive framework:

- **Establish or advance consensus definitions and prioritized contexts of use (COUs)** for psychiatric biomarkers, aligned with FDA regulatory nomenclature (e.g., predictive, stratification, surrogate, and safety biomarkers), and to address ambiguity (highlighted in *Sections “Definitions and Context of Use”* and *“Regulatory Gaps”)*. This effort should explicitly distinguish exploratory discovery from validation-ready applications to reduce downstream regulatory and commercial risk. As a leading convener of scientific and translational stakeholders, ACNP is well-positioned to initiate structured dialogues with regulatory agencies (e.g., through workshops or joint advisory forums) aimed at harmonizing terminology, evidentiary expectations, and COU definitions across consortia.
- **Prioritize small-scale, prospective biomarker studies using mechanism-engaged and within-subject designs in anticipation of future large-scale studies**, (as outlined in *Section “Trial Design Frameworks”)*, including crossover or short-term intervention paradigms (e.g., fast-acting probes). These studies should focus on scalable and pharmacodynamically sensitive modalities—particularly *fluid biomarkers and genomics, digital phenotyping, and EEG*—to maximize signal detection while maintaining operational feasibility, and to ensure appropriate participant stratification.
- **Improve preclinical experimental systems** by ensuring translational relevance, focusing whenever possible on procedures and biomarkers that can be directly compared across species. Given the ubiquitous availability of personal devices that can track health-related metrics and their increasing utilization in the healthcare ecosystem, the use of digital biomarkers (e.g., sleep architecture, heart and respiratory rates, circadian rhythms of body temperature and locomotor activity) that are altered in neuropsychiatric disorders should be prioritized. Improvements in the predictive validity of experimental systems across levels of analyses and species, where appropriate, for human outcomes, identified at the earliest stages of therapeutic development, will help to avoid protracted and expensive translational failures.
- **Systematically leverage retrospective validation across existing large cohorts and trials**, including EMBARC, ABC-CT, EU-AIMS/LEAP, AMP-SCZ, and related datasets (*Sections “Real-World Case Studies” and “Trial Design Frameworks”*), to test generalizability, replication, and boundary conditions of candidate biomarkers before launching costly prospective trials. Emphasis should be placed on validation rather than novel discovery.
- **Develop a precompetitive data-sharing, governance, and legal framework**, modeled on established entities such as the Critical Path Institute [105, 106] to support harmonized protocols, pooled analyses, and shared negative as well as positive findings (*Sections “De-risking Strategies” and “Integration with Ongoing Initiatives”*). This framework should explicitly address data standards, intellectual property boundaries, and analytic transparency to enable broad participation from academia and industry. This framework should support scalability from small proof-of-concept studies to larger consortia trials, include structures for periodic updating of biomarker repositories based on evolving evidence, and establish agreed-upon held-out datasets to enable truly independent validation and replication across platforms and populations, as well as guidelines on informed consent documents that permit sharing of relevant clinical trial data elements. Key elements should include shared metadata standards, reference assays, and clear IP ownership and data access guidelines to ensure broad participation across academia and industry.
- **Implement standardized acquisition, quality control, and analytic pipelines across biomarker modalities**, building on lessons from ADNI and other neurological consortia (*Sections “Candidate Biomarker Modalities” and “Derisking Strategies”*). CDEs, reference assays, and device standards should be prioritized to improve reproducibility and cross-study comparability.
- **Engage regulatory agencies early and iteratively**—including FDA and EMA—through precompetitive consortia or agency-specific engagement pathways (e.g., CPIM, ISTAND, parallel scientific advice) to review proposed biomarker frameworks, clarify evidentiary thresholds, and provide feedback on acceptable pathways for IND inclusion versus formal qualification (*Sections “Regulatory Landscape” and “Expanded Regulatory Landscape”*). Early dialogue should explicitly address composite, multimodal, and AI-derived biomarkers.
- **Align stakeholder incentives across academia, industry, regulators, and payers** by demonstrating how biomarker-enabled trial designs can reduce development costs, improve signal detection, and support value-based care (*Section “Stakeholder Incentives and Translational Levers”*). Health-economic modeling and real-world feasibility studies should be incorporated early to strengthen the case for adoption beyond proof-of-concept.

Together, these recommendations outline a pragmatic, stepwise pathway for transitioning psychiatric biomarkers from exploratory signals to validated, regulatory-interpretable tools. By explicitly mapping scientific strategy to regulatory expectations and embedding biomarker development within precompetitive, collaborative infrastructures, the field can accelerate progress toward precision psychiatry while minimizing duplication, uncertainty, and translational failure.

## Conclusions: advancing a precompetitive precision biomarker ecosystem in psychiatry

Psychiatry stands at a pivotal moment. The scientific tools needed to define biologically grounded, scalable biomarkers—spanning fluid assays and genomic biomarkers, digital phenotyping, electrophysiology, neuroimaging, and multimodal analytics—are now available, yet their translation into predictable mechanistic biomarkers and targets, clinical trials and regulatory decision-making remains fragmented. As outlined in this roadmap, *the principal barriers are no longer purely technical, they are also structural and systemic* and include: misaligned incentives, unclear contexts of use, insufficient standardization, and the absence of sustained precompetitive infrastructure.

We therefore call on academic investigators, industry sponsors, regulators, and funders to commit to a *coordinated, precompetitive strategy for biomarker development in psychiatry*. This includes converging on shared definitions and use cases, investing in small but rigorous prospective studies, systematically validating biomarkers across existing large cohorts, and engaging regulators early to shape feasible evidentiary pathways. Importantly, success will require valuing replication, negative findings, and harmonization as highly as novelty.

By adopting this collective approach, modeled on successful frameworks in neurodegenerative disease, but tailored to the unique complexity of psychiatric disorders, the field can transition from exploratory signals to regulatory-ready tools that meaningfully improve trial efficiency, therapeutic targeting, and patient outcomes. Precision psychiatry will not emerge from isolated breakthroughs, but from a shift to sustained collaboration around shared standards, shared data, and shared responsibility for translation and implementation.

### Citation diversity statement

The authors have attested that they made efforts to be mindful of diversity in selecting the citations used in this article.
